# Supplementary figures and images for: Hyperglycemia induced cathepsin L maturation linked to diabetic comorbidities and COVID-19 mortality
Source: eLife. 2024 Aug 16;13:RP92826. doi: 10.7554/eLife.92826 (PMC11329274; doi:10.7554/eLife.92826)

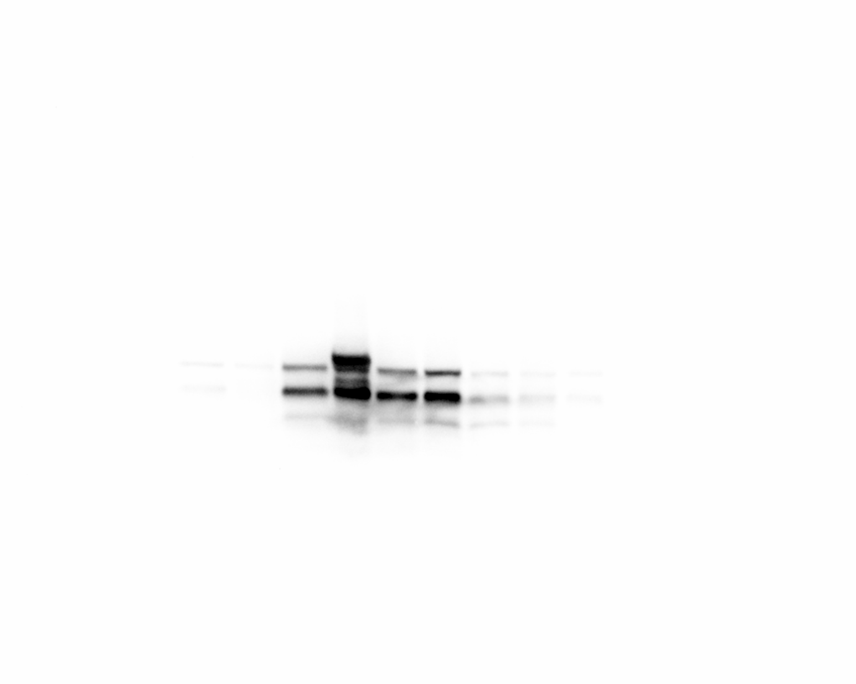

Supplement: Figure 3—source data 2. [file elife-92826-fig3-data2.zip › Figure3SourceData2/Figure 3 CTSL.tif]

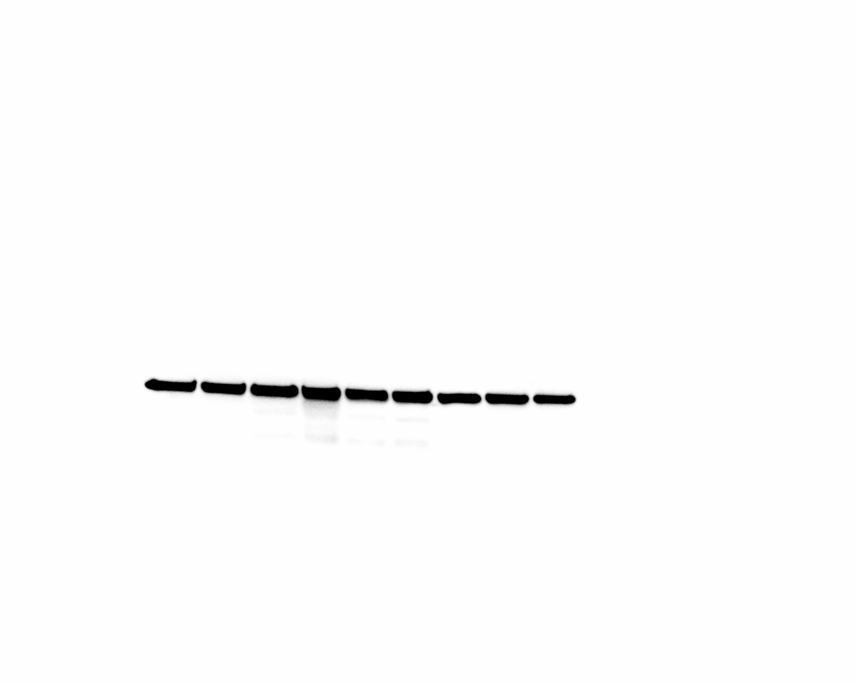

Supplement: Figure 3—source data 2. [file elife-92826-fig3-data2.zip › Figure3SourceData2/Figure 3 Tubulin.tif]

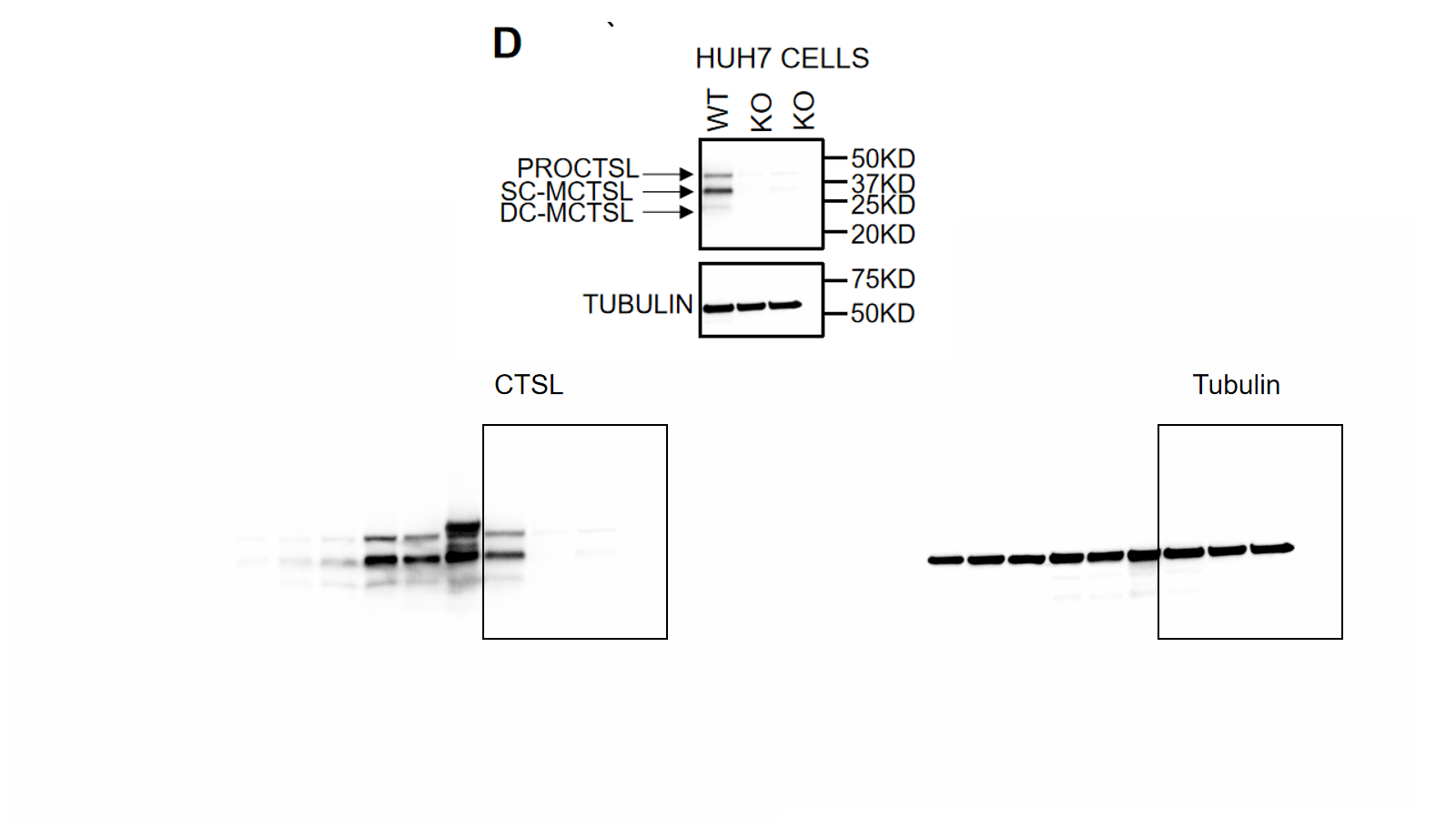

Supplement: Figure 3—source data 2. [file elife-92826-fig3-data2.zip › Figure3SourceData2/Fig 3D_Labelled.tif]

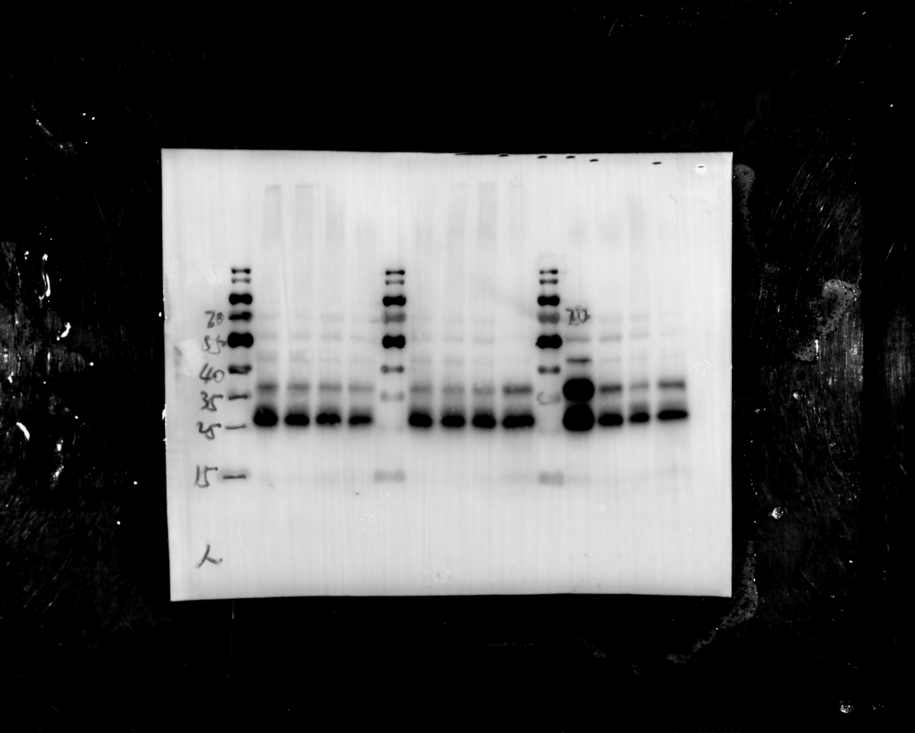

Supplement: Figure 5—source data 2. [file elife-92826-fig5-data2.zip › Figure5SourceData2/Figure 5E CTSL.tif]

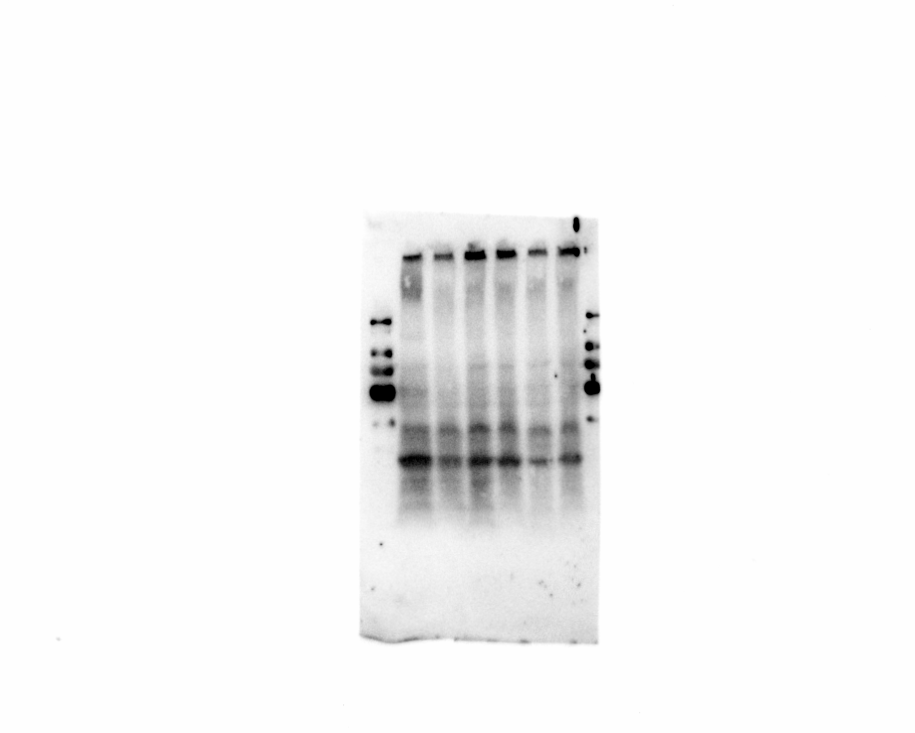

Supplement: Figure 5—source data 2. [file elife-92826-fig5-data2.zip › Figure5SourceData2/Figure 5D CTSL.tif]

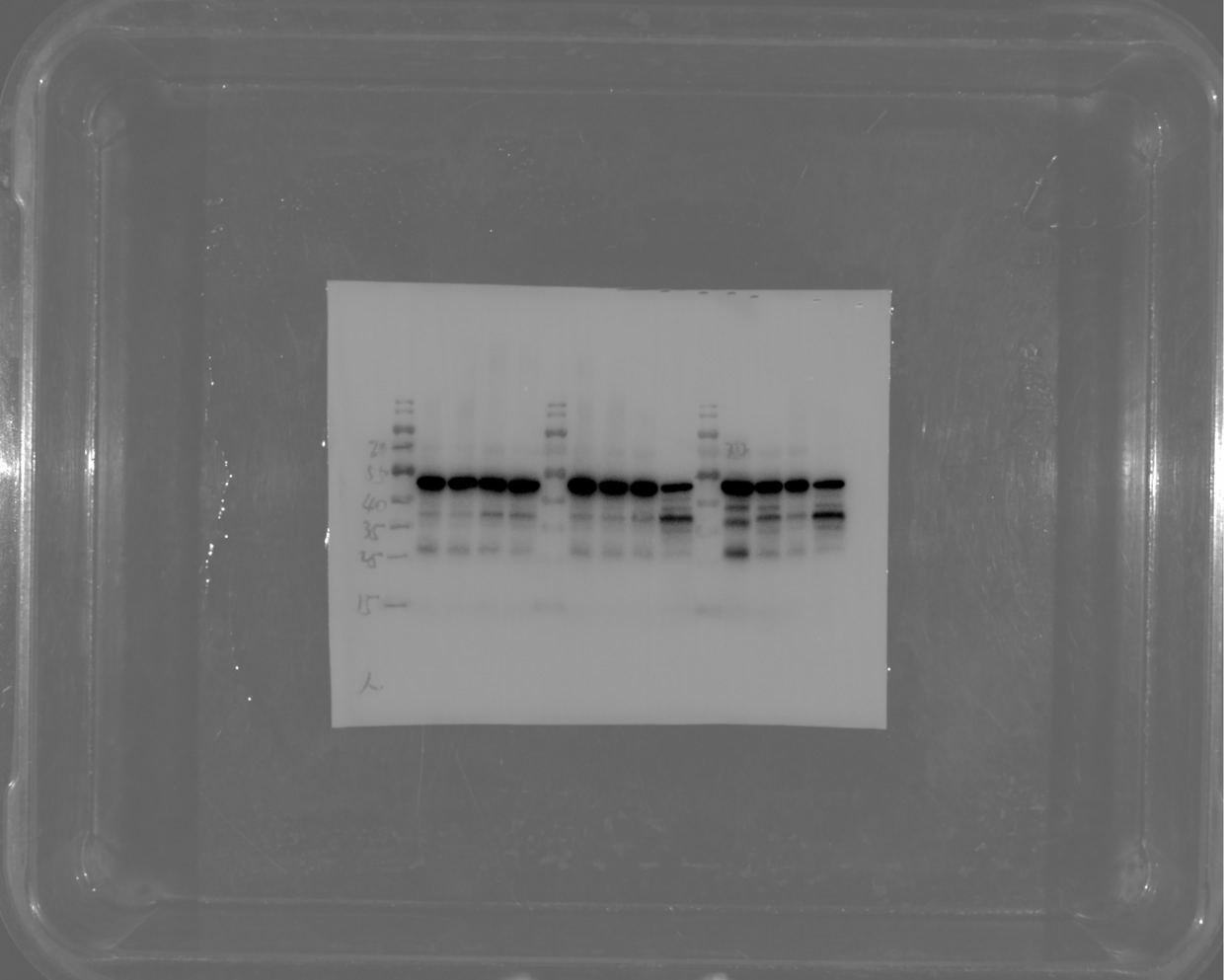

Supplement: Figure 5—source data 2. [file elife-92826-fig5-data2.zip › Figure5SourceData2/Figure 5E Actin.tif]

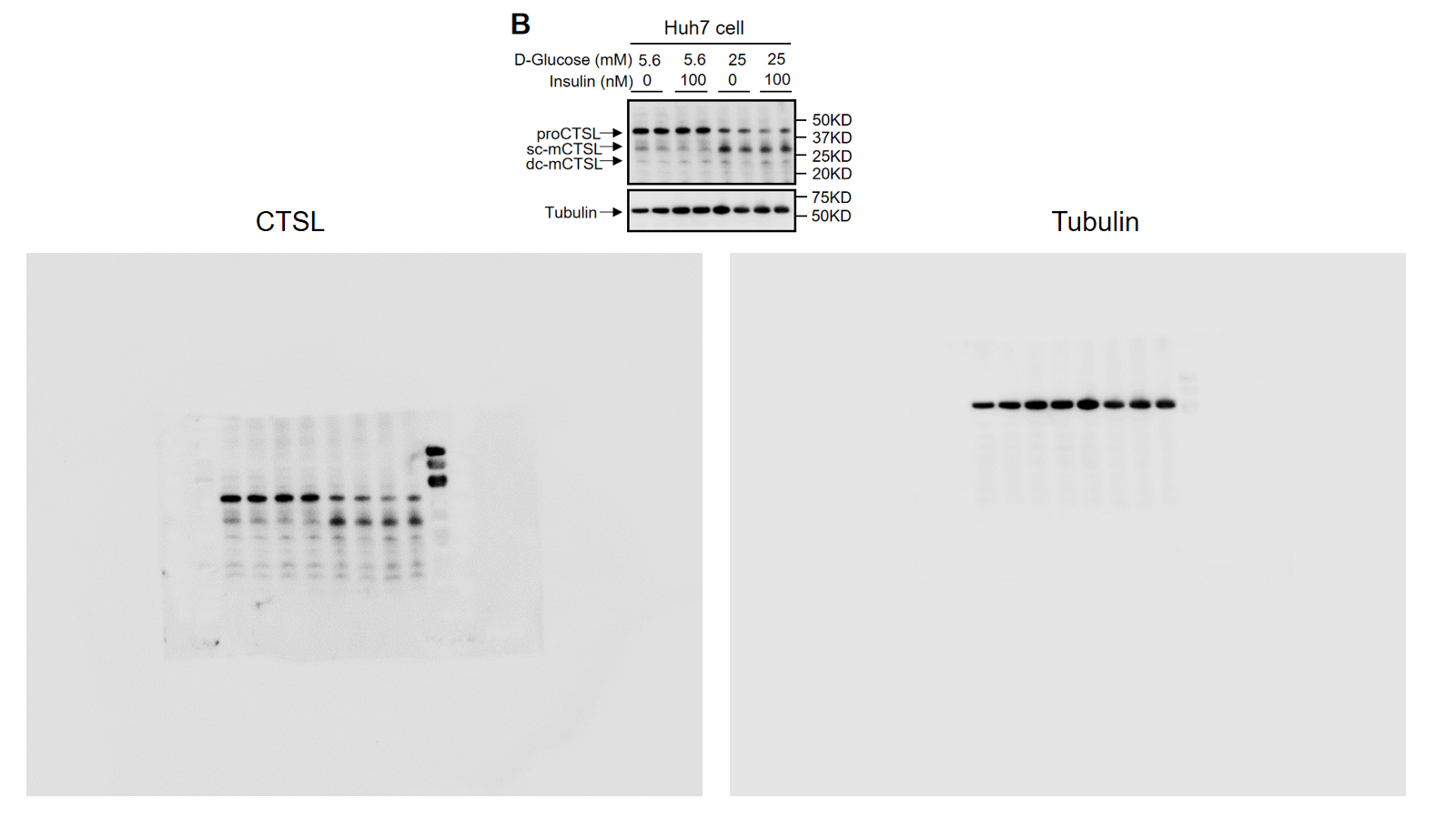

Supplement: Figure 5—source data 2. [file elife-92826-fig5-data2.zip › Figure5SourceData2/Fig 5B_Labelled.tif]

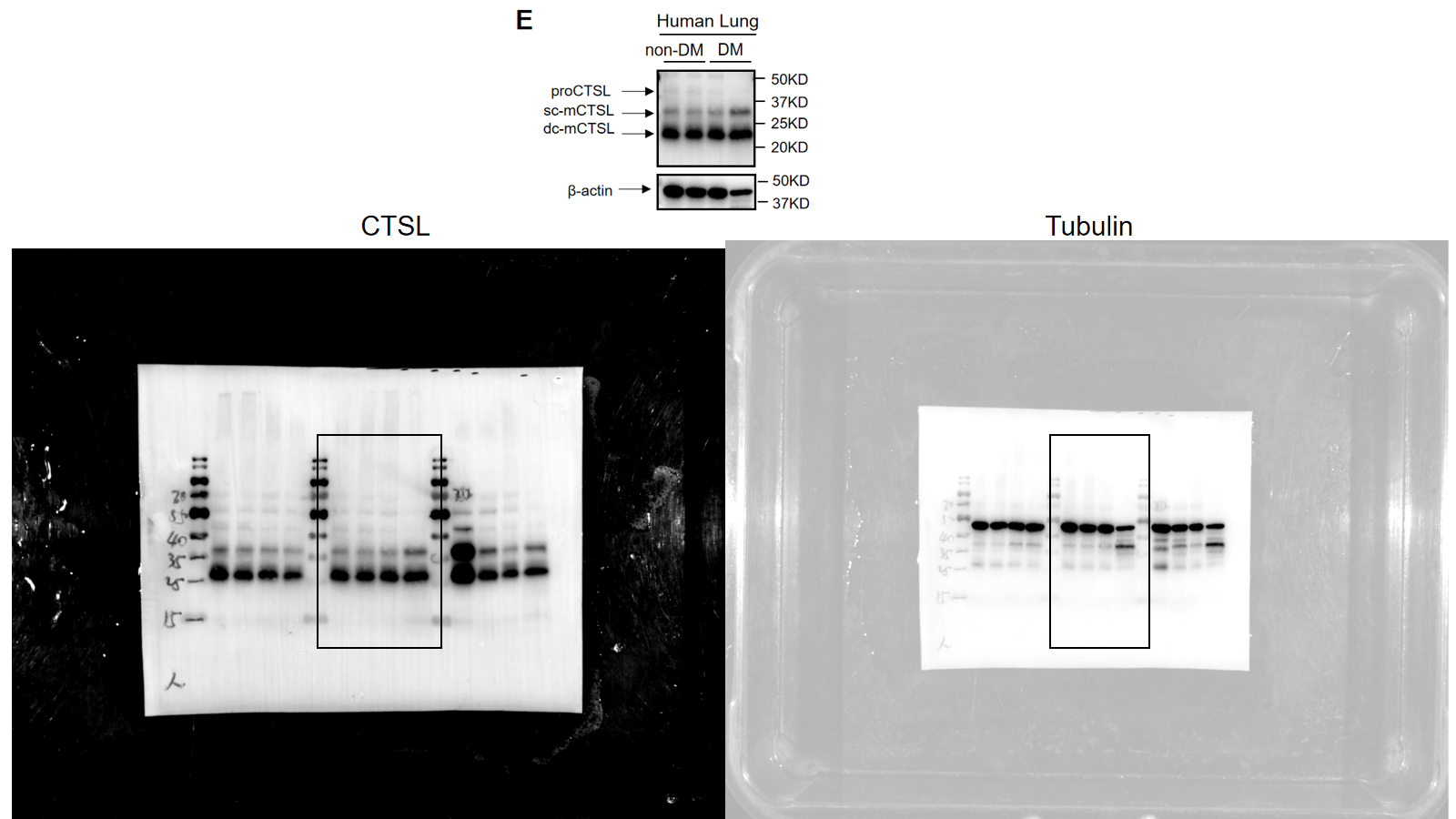

Supplement: Figure 5—source data 2. [file elife-92826-fig5-data2.zip › Figure5SourceData2/Fig 5E_Labelled.tif]

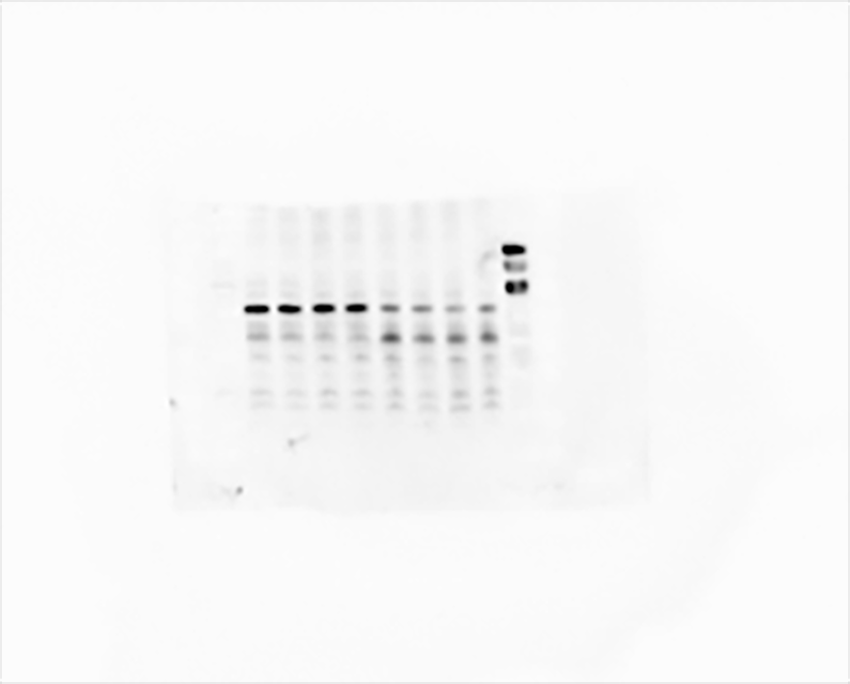

Supplement: Figure 5—source data 2. [file elife-92826-fig5-data2.zip › Figure5SourceData2/Figure 5B CTSL.tif]

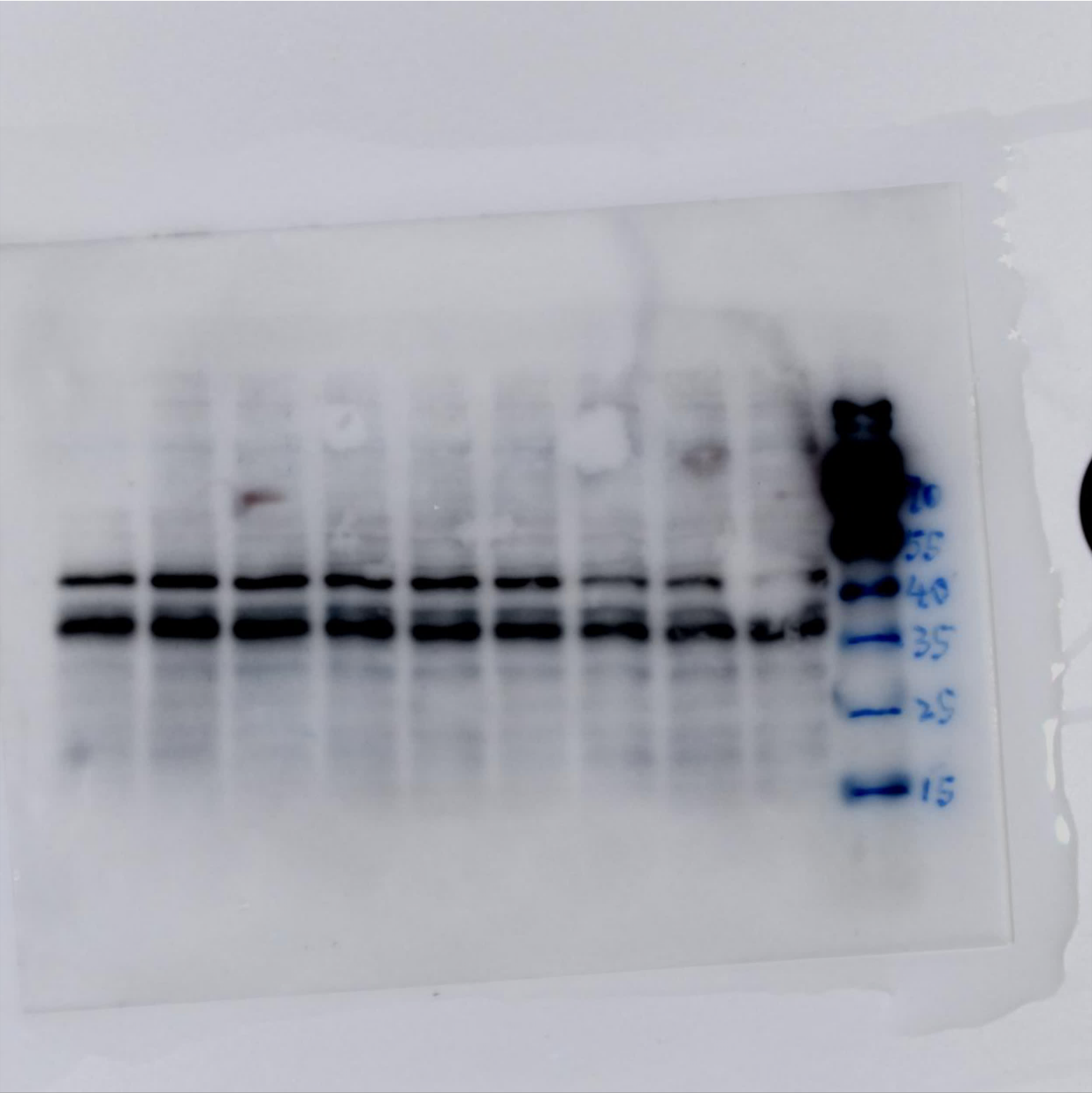

Supplement: Figure 5—source data 2. [file elife-92826-fig5-data2.zip › Figure5SourceData2/Figure 5C CTSL.tif]

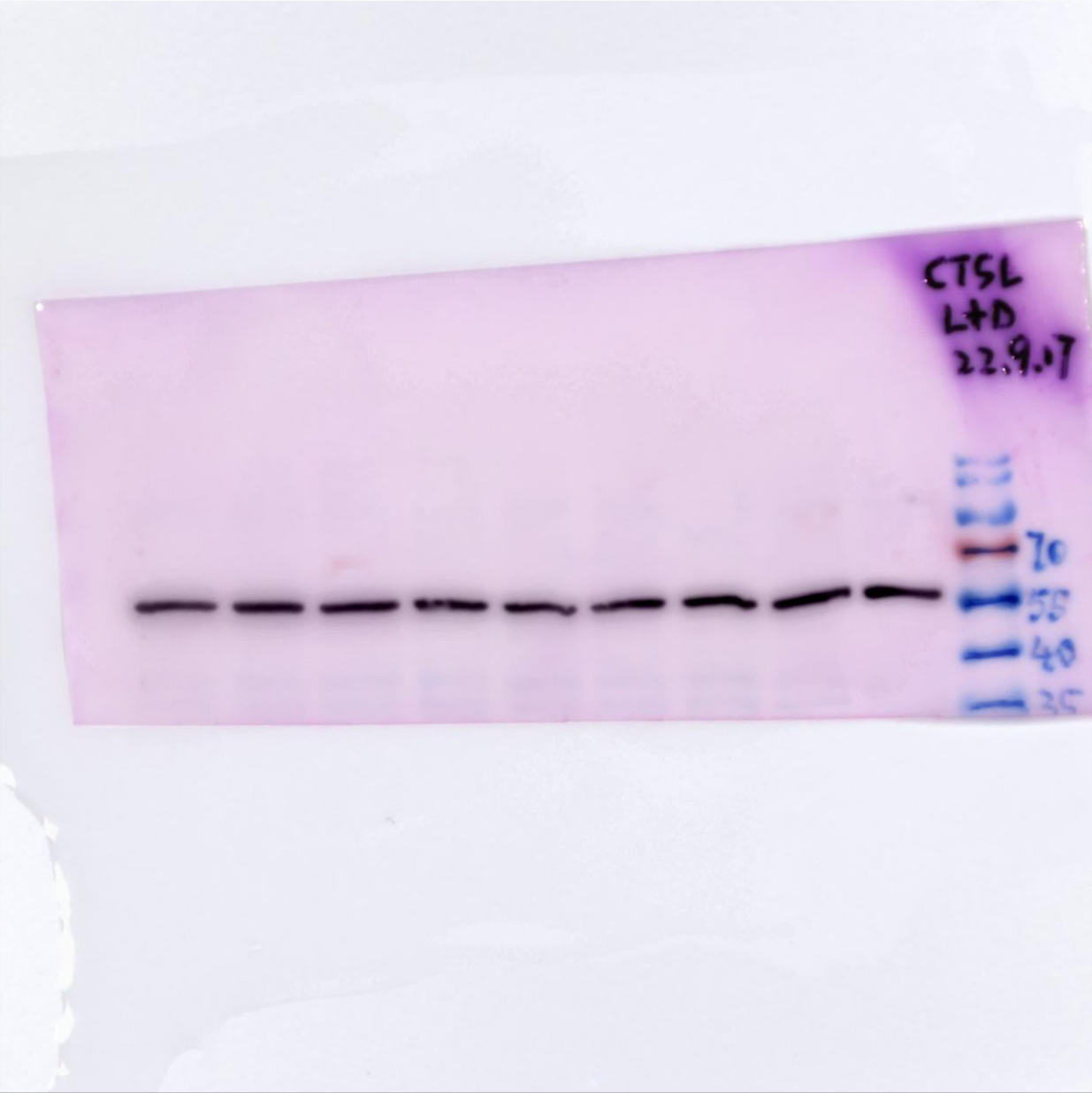

Supplement: Figure 5—source data 2. [file elife-92826-fig5-data2.zip › Figure5SourceData2/Figure 5C Tubulin.tif]

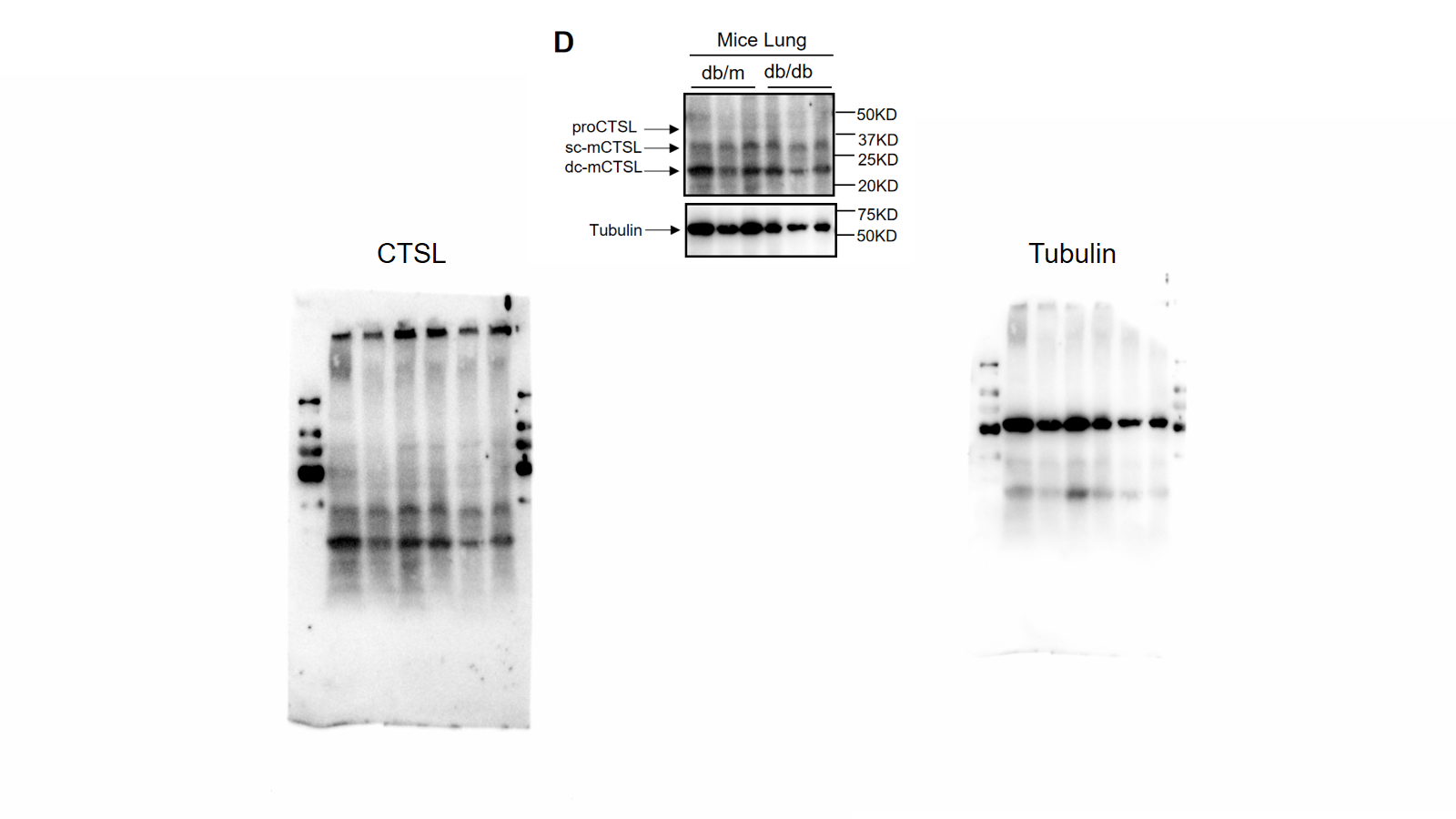

Supplement: Figure 5—source data 2. [file elife-92826-fig5-data2.zip › Figure5SourceData2/Fig 5D_Labelled.tif]

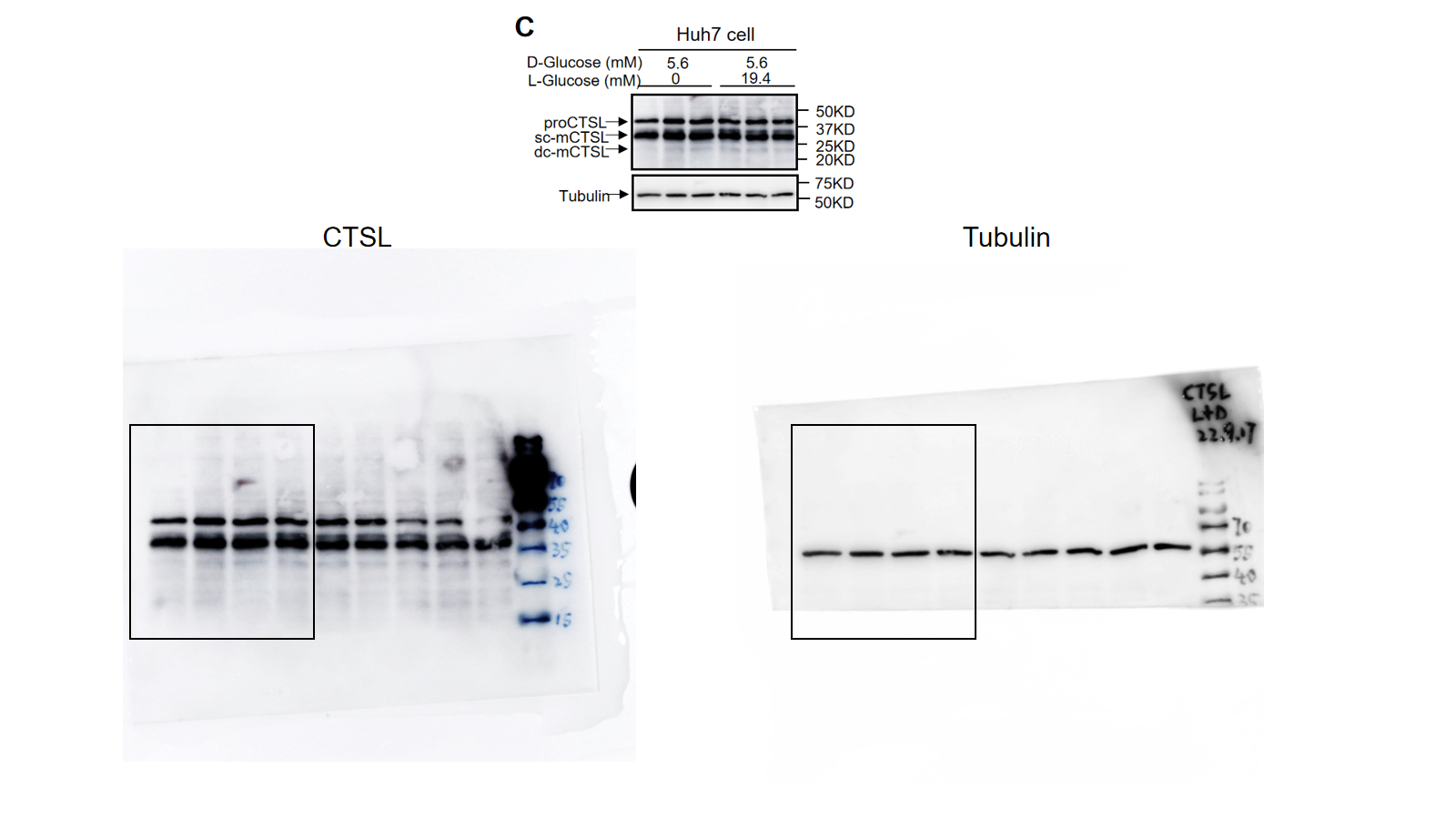

Supplement: Figure 5—source data 2. [file elife-92826-fig5-data2.zip › Figure5SourceData2/Fig 5C_Labelled.tif]

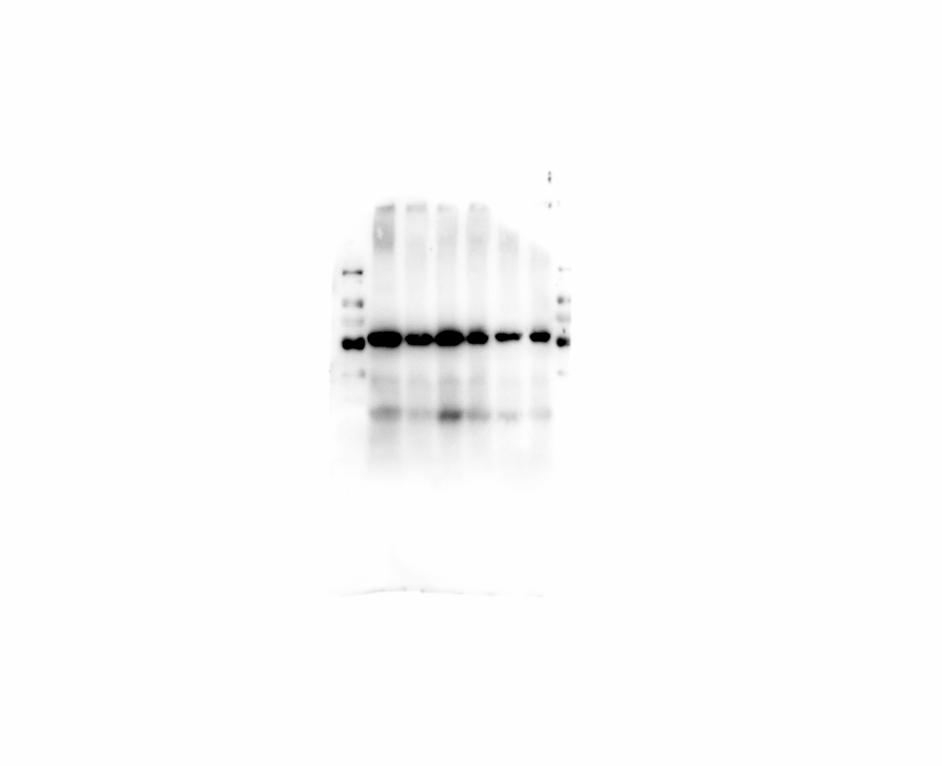

Supplement: Figure 5—source data 2. [file elife-92826-fig5-data2.zip › Figure5SourceData2/Figure 5D Tubulin.tif]

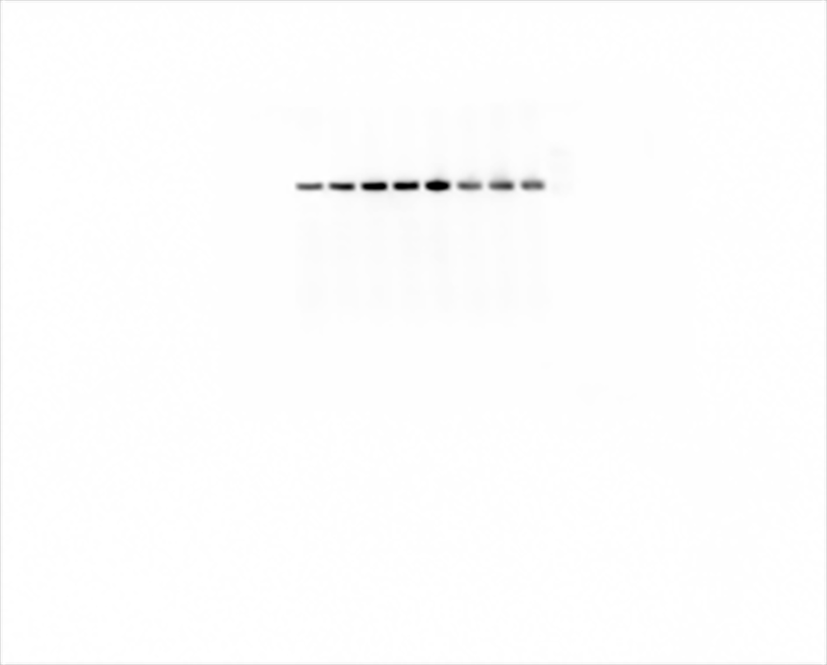

Supplement: Figure 5—source data 2. [file elife-92826-fig5-data2.zip › Figure5SourceData2/Figure 5B Tubulin.tif]

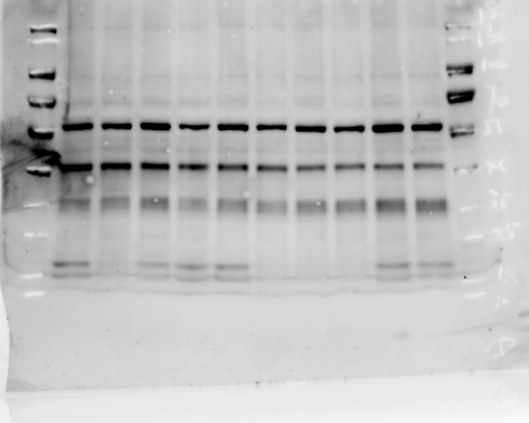

Supplement: Figure 5—figure supplement 2—source data 2. [file elife-92826-fig5-figsupp2-data2.zip › Figure5S1SourceData2/Figure 5-figure supplement 2-Tubulin.tif]

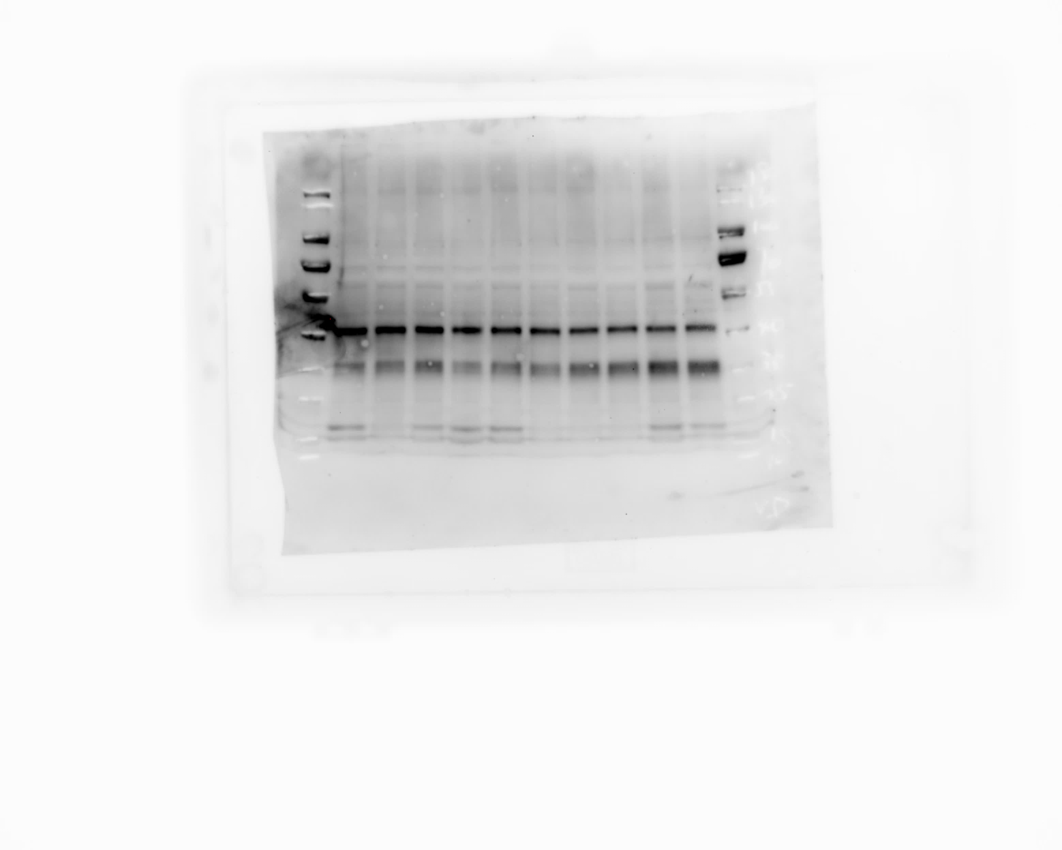

Supplement: Figure 5—figure supplement 2—source data 2. [file elife-92826-fig5-figsupp2-data2.zip › Figure5S1SourceData2/Figure 5-figure supplement 2-CTSL.tif]

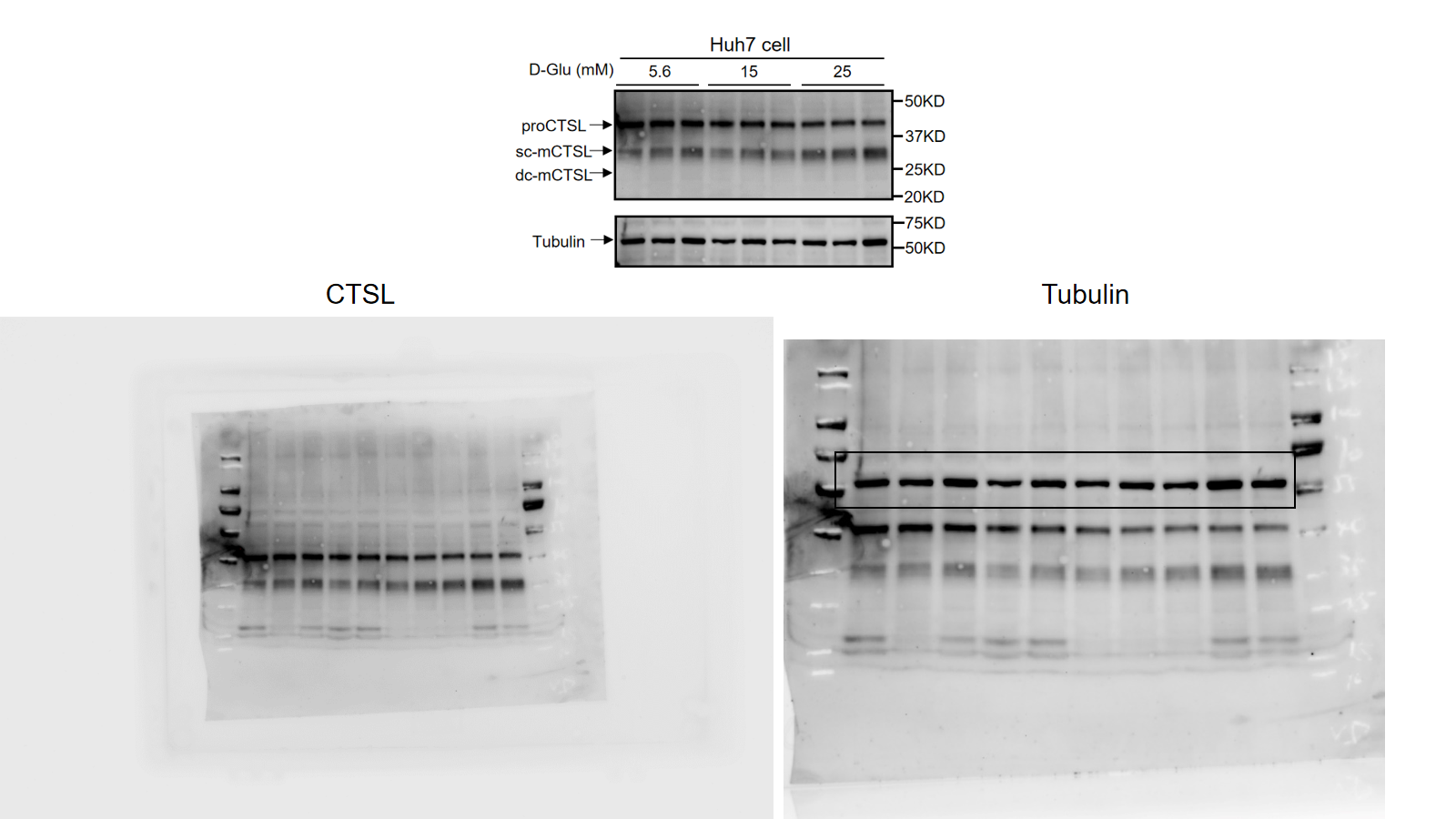

Supplement: Figure 5—figure supplement 2—source data 2. [file elife-92826-fig5-figsupp2-data2.zip › Figure5S1SourceData2/Fig 5-supplement 2_labelled.tif]
