# Supplementary material for: Hyperglycemia induced cathepsin L maturation linked to diabetic comorbidities and COVID-19 mortality
Source: eLife. 2024 Aug 16;13:RP92826. doi: 10.7554/eLife.92826 (PMC11329274; doi:10.7554/eLife.92826)
Supplement: Supplementary file 1. — Data are median (IQR) or n (%). P values were calculated by Mann-Whitney U-test (†) or χ² test (#), as appropriate for group comparison analyses. [file elife-92826-supp1.docx]

|  | All patients  (n = 62) | COVID-19 | | |
| --- | --- | --- | --- | --- |
|  |  | DM  (n = 31) | Non-DM  (n = 31) | *P* value |
| Age—years | 60 (52-67) | 57 (51-65) | 61 (56-70) | 0.212† |
| Male—n (%) | 31 (50%) | 19 (61.3%) | 12 (38.7%) | 0.075# |
| Hemoglobin—g/L | 129 (117-142) | 123 (114-135) | 135 (120-145) | 0.090† |
| Platelet count— × 10^9^/L | 211 (186-289) | 205 (161-278) | 221 (206-293) | 0.109† |
| White blood cell count— × 10^9^/L | 6.04 (4.60-7.94) | 6.09 (4.82-8.03) | 5.95 (4.47-7.70) | 0.469† |
| Neutrophil percent—% | 71.6 (65.6-84.4) | 77.6 (67.5-89.0) | 68.2 (65.3-73.5) | **0.007**† |
| Lymphocyte percent—% | 18.7 (11.4-27.2) | 14.5 (7.0-21.0) | 21.7 (17.1-28.1) | **0.006**† |
| Potassium—mmol/L | 4.03 (3.55-4.41) | 3.98 (3.52-4.38) | 4.04 (3.58-4.43) | 0.897† |
| Sodium—mmol/L | 138.8 (135.7-141.4) | 136.3 (135.0-140.3) | 139.8 (138.1-142.0) | **0.005**† |
| Chloride—mmol/L | 102.5 (99.1-105.4) | 100.7 (98.3-104.2) | 104.2 (100.8-106.0) | **0.029**† |
| Albumin—g/L | 29.4 (27.3-35.0) | 28.5 (25.6-34.1) | 31.6 (27.6-35.7) | 0.202† |
| Albumin/Globulin ratio | 0.90 (0.70-1.27) | 0.83 (0.70-1.08) | 1.05 (0.80-1.33) | **0.013**† |
| C-reactive protein—mg/L | 29.0 (4.1-70.8) | 53.4 (16.1-91.9) | 11.3 (2.0-40.3) | **0.012**† |
| Procalcitonin—mg/L | 0.08 (0.05-0.16) | 0.12 (0.07-0.23) | 0.06 (0.04-0.10) | **0.013**† |
| Alanine aminotransferase—U/L | 38 (24-50) | 40 (28-53) | 34 (22-44) | 0.105† |
| Aspartate aminotransferase—U/L | 31 (22-47) | 34 (27-52) | 29 (19-40) | **0.037**† |
| Creatine kinase–MB—ng/mL | 0.44 (0.21-0.88) | 0.75 (0.43-1.68) | 0.32 (0.18-0.63) | **0.006**† |
| Myoglobin—g/L | 47.1 (28.0-75.0) | 60.4 (39.4-129.5) | 33.5 (26.1-51.9) | **0.002**† |
| Blood urea nitrogen—mmol/L | 5.03 (3.41-6.78) | 5.79 (4.31-7.49) | 4.10 (3.11-5.47) | **0.019**† |
| Creatinine—μmol/L | 68.3 (56.9-86.6) | 74.0 (60.0-94.2) | 63.5 (52.2-73.4) | **0.017**† |

**Supplementary File 1. Comparation of clinical features of COVID-19 patients**

Data are median (IQR) or n (%). *P* values were calculated by Mann-Whitney *U*-test (†) or χ² test (#), as appropriate for group comparison analyses.
