# Supplementary material for: Hyperglycemia induced cathepsin L maturation linked to diabetic comorbidities and COVID-19 mortality
Source: eLife. 2024 Aug 16;13:RP92826. doi: 10.7554/eLife.92826 (PMC11329274; doi:10.7554/eLife.92826)
Supplement: Supplementary file 2. — Data are median (IQR) or n (%). P values were calculated by Mann-Whitney U-test (†) or χ² test (#), as appropriate for group comparison analyses. [file elife-92826-supp2.docx]

**Supplementary File 2. Demographic and clinical characteristics of non-COVID-19 patients**

|  | All individuals  N = 122 | Non-COVID-19 | | |
| --- | --- | --- | --- | --- |
|  |  | Healthy  N = 61 | DM  N = 61 | *P* |
| Age—years | 59 (55-62) | 58 (55-64) | 60 (58-62) | 0.141† |
| Male—n (%) | 58 (47.5%) | 27 (44.3%) | 31 (50.8%) | 0.468# |
| BMI | 25.01 (22.37-27.34) | 24.8 (22.2-26.7) | 25.4 (22.8-27.5) | 0.347† |
| HbA1c—% | 5.85 (5.30-7.13) | 5.4 (5.2-5.8) | 7.0 (6.2-8.2) | **0.000**† |
| CTSL conc. —pg/mL | 1637.0 (1337.8-2120.9) | 1535.4 (1251.3-1838.7) | 1715.7 (1408.0-2261.4) | **0.009**† |
| CTSL activity—RLU | 1708.0 (477.2-3058.7) | 477.2 (429.8-601.8) | 3050.0 (2534.0-3713.0) | **0.000**† |

Data are median (IQR) or n (%). *P* values were calculated by Mann-Whitney *U-*test (†) or χ² test (#), as appropriate for group comparison analyses.
