# Supplementary material for: Hyperglycemia induced cathepsin L maturation linked to diabetic comorbidities and COVID-19 mortality
Source: eLife. 2024 Aug 16;13:RP92826. doi: 10.7554/eLife.92826 (PMC11329274; doi:10.7554/eLife.92826)
Supplement: Supplementary file 3. — Data are correlation coefficient (P value). Spearman’s rho test (two-tailed). [file elife-92826-supp3.docx]

**Supplementary File** **3. Nonparametric correlations of parameters correlated with CTSL levels and diabetes in non-COVID-19 individuals**

|  | CTSL activity | CTSL conc | HbA1c | BMI | Systolic pressure | Diastolic pressure | | Waist | Hip | WHR | LDL | Serum CREA | Age | Gender | Diabetes | HBP | CHD |
| --- | --- | --- | --- | --- | --- | --- | --- | --- | --- | --- | --- | --- | --- | --- | --- | --- | --- |
| CTSL activity | 1.00 |  |  |  |  |  |  | |  |  |  |  |  |  |  |  |  |
| CTSL  conc | **0.248**  **(0.006)** | 1.00 |  |  |  |  |  | |  |  |  |  |  |  |  |  |  |
| HbA1c | **0.565**  **(0.000)** | **0.263**  **(0.003)** | 1.00 |  |  |  |  | |  |  |  |  |  |  |  |  |  |
| BMI | 0.162  (0.084) | 0  (0.997) | 0.134  (0.152) | 1.00 |  |  |  | |  |  |  |  |  |  |  |  |  |
| Systolic pressure | **0.314**  **(0.001)** | **0.415**  **(0.000)** | **0.294**  **(0.002)** | 0.182  (0.056) | 1.00 |  |  | |  |  |  |  |  |  |  |  |  |
| Diastolic  pressure | **-0.235**  **(0.012)** | 0.048  (0.613) | -0.124  (0.190) | **0.232**  **(0.014)** | **0.213**  **(0.023)** | 1.00 |  | |  |  |  |  |  |  |  |  |  |
| Waist | 0.045  (0.634) | 0.075  (0.421) | 0.139  (0.138) | **0.728**  **(0.000)** | **0.204**  **(0.031)** | **0.191**  **(0.044)** | 1.00 | |  |  |  |  |  |  |  |  |  |
| Hip | **-0.246**  **(0.008)** | -0.023  (0.809) | -0.038  (0.688) | **0.572**  **(0.000)** | 0.120  (0.208) | **0.284**  **(0.002)** | **0.678**  **(0.000)** | | 1.00 |  |  |  |  |  |  |  |  |
| WHR | **0.385**  **(0.000)** | 0.115  (0.222) | **0.241**  **(0.009)** | **0.331**  **(0.000)** | **0.198**  **(0.037)** | -0.031  (0.744) | **0.525**  **(0.000)** | | -0.157  (0.093) | 1.00 |  |  |  |  |  |  |  |
| LDL | **-0.264**  **(0.003)** | 0.066  (0.468) | -0.058  (0.526) | -0.011  (0.903) | -0.090  (0.339) | **0.202**  **(0.031)** | 0.007  (0.941) | | 0.089  (0.342) | -0.098  (0.295) | 1.00 |  |  |  |  |  |  |
| Serum CREA | -0.096  (0.291) | **0.189**  **(0.037)** | -0.102  (0.262) | 0.041  (0.664) | 0.071  (0.450) | 0.103  (0.277) | 0.177  (0.058) | | 0.131  (0.163) | 0.070  (0.458) | -0.066  (0.467) | 1.00 |  |  |  |  |  |
| Age | **0.205**  **(0.023)** | **0.261**  **(0.004)** | **0.233**  **(0.010)** | 0.028  (0.762) | **0.211**  **(0.024)** | -0.065  (0.492) | 0.085  (0.362) | | 0.045  (0.631) | 0.122  (0.196) | 0.065  (0.479) | -0.046  (0.618) | 1.00 |  |  |  |  |
| Gender | 0.061  (0.504) | **0.322**  **(0.000)** | 0.023  (0.801) | -0.028  (0.768) | 0.086  (0.365) | 0.077  (0.416) | 0.148  (0.112) | | 0.081  (0.392) | 0.094  (0.317) | -0.142  (0.118) | **0.528**  **(0.000)** | **0.217**  **(0.016)** | 1.00 |  |  |  |
| Diabetes | **0.866**  **(0.000)** | **0.239**  **(0.008)** | **0.669**  **(0.000)** | 0.088  (0.350) | **0.348**  **(0.000)** | **-0.221**  **(0.018)** | -0.003  (0.978) | | **-0.309**  **(0.001)** | **0.389**  **(0.000)** | **-0.270**  **(0.003)** | -0.046  (0.618) | **0.254**  **(0.005)** | 0.066  (0.472) | 1.00 |  |  |
| HBP | **0.676**  **(0.000)** | **0.188**  **(0.038)** | **0.593**  **(0.000)** | 0.174  (0.063) | **0.397**  **(0.000)** | -0.131  (0.164) | 0.077  (0.414) | | -0.167  (0.075) | **0.303**  **(0.001)** | **-0.214**  **(0.018)** | -0.087  (0.342) | **0.233**  **(0.010)** | -0.057  (0.532) | **0.829**  **(0.000)** | 1.00 |  |
| CHD | **0.445**  **(0.000)** | **0.179**  **(0.049)** | **0.350**  **(0.000)** | **0.265**  **(0.004)** | **0.265**  **(0.004)** | -0.037  (0.699) | **0.200**  **(0.031)** | | -0.040  (0.672) | **0.383**  **(0.000)** | -0.164  (0.071) | 0.090  (0.324) | **0.214**  **(0.018)** | 0.046  (0.615) | **0.533**  **(0.000)** | **0.579**  **(0.000)** | 1.00 |

Data are correlation coefficient (*P* value). Spearman's rho test (two-tailed).
