# Supplementary material for: Hyperglycemia induced cathepsin L maturation linked to diabetic comorbidities and COVID-19 mortality
Source: eLife. 2024 Aug 16;13:RP92826. doi: 10.7554/eLife.92826 (PMC11329274; doi:10.7554/eLife.92826)
Supplement: Supplementary file 4. — Six enrolled patients undergoing lung surgery in general surgery department of Beijing Tongren Hospital ranging from March 22 to June 22, 2022. [file elife-92826-supp4.docx]

**Supplementary File 4. Demographic and clinical characteristics of human lung tissues donor**

|  | No. | Gender | Glucose-mmol/L | LDL-C-mmol/L |
| --- | --- | --- | --- | --- |
| DM | 1 | Male | 5.2 | 3.31 |
|  | 2 | Female | 5.6 | 2.49 |
|  | 3 | Male | 8 | 2.45 |
| Non-DM | 4 | Male | 6 | 1.64 |
|  | 5 | Female | 5.2 | 3.98 |
|  | 6 | Male | 4.2 | 4.28 |

Six enrolled patients undergoing lung surgery in general surgery department of Beijing Tongren Hospital ranging from March 22 to June 22, 2022.
